# Correlation between Blood Activin Levels and Clinical Parameters of Type 2 Diabetes

**DOI:** 10.1155/2012/410579

**Published:** 2012-12-16

**Authors:** Hui Wu, Michael Wu, Yi Chen, Carolyn A. Allan, David J. Phillips, Mark P. Hedger

**Affiliations:** ^1^Monash Institute of Medical Research, Monash University, Clayton, VIC 3168, Australia; ^2^School of Public Health, Monash University, Clayton, VIC 3168, Australia; ^3^Prince Henry's Institute, Melbourne, VIC 3168, Australia; ^4^Paranta Biosciences, Richmond, VIC 3121, Australia

## Abstract

*Aims*. Activins A and B, and their binding protein, follistatin, regulate glucose metabolism and inflammation. Consequently, their role in type 2 diabetes (T2D) was examined. *Methods*. Blood was taken from fasted participants (34 males; 58 females; 50–75 years) with diabetes or during an oral glucose tolerance test (OGTT). Clinical parameters were assessed, and blood assayed for activins, follistatin, and C-reactive protein. *Results*. Serum levels of activin A (93.3 ± 27.0 pg/mL, mean ± SD), B (81.8 ± 30.8 pg/mL), or follistatin (6.52 ± 3.15 ng/mL) were not different (*P* > 0.05) between subjects with normal OGTT (*n* = 39), impaired glucose tolerance and/or fasting glucose (*n* = 17), or T2D (*n* = 36). However, activin A and/or activin B were positively correlated with parameters of insulin resistance and T2D, including fasting glucose (*P* < 0.001), fasting insulin (*P* = 0.02), glycated hemoglobin (*P* = 0.003), and homeostasis model assessment of insulin resistance (HOMA-IR; *P* < 0.001). Follistatin was positively correlated with HOMA-IR alone (*P* = 0.01). *Conclusions*. These data indicate that serum measurements of activin A, B, or follistatin cannot discriminate risk for T2D in individual patients, but the activins display a positive relationship with clinical parameters of the disease.

## 1. Introduction

Type 2 diabetes (T2D) is a metabolic disorder with chronic inflammation characterised by insulin resistance and hyperglycaemia. Obesity-induced inflammation has been implicated as an important aetiological factor in the development of insulin resistance and onset of T2D [1, 2]. Excessive weight gain leads to increased macrophage infiltration of white adipose tissue, and increased central adiposity is associated with a chronic inflammatory state, induced by proinflammatory mediators, such as tumor necrosis factor (TNF), interleukin 6 (IL-6), and C-reactive protein (CRP) [3, 4]. Inflammatory mediators may also be increased in T2D and insulin resistance, and inhibit insulin signaling through activation of Jun N-terminal kinase (JNK) and nuclear factor (NF)-*κ*B [5, 6], thereby causing insulin resistance and interfering with glucose homeostasis [2, 7, 8]. TNF mRNA is more highly expressed in the fat of obese premenopausal women than that of lean controls, and TNF mRNA expression levels show a positive correlation with hyperinsulinaemia [9]. Furthermore, anti-inflammatory agents such as aspirin or anti-TNF are useful in the control of T2D [10, 11].

Activins A and B are members of the transforming growth factor-*β* (TGF-*β*) superfamily and are elevated in the blood and tissues in a broad range of inflammatory diseases [12]. Activin A has long been known to be a critical regulator of inflammation that, like TNF, is upregulated early in inflammation [12, 13]. A potential role for activin B in inflammatory disease has become apparent only more recently. The biological activity of both activins is regulated by a high-affinity activin-binding protein, called follistatin [13]. 

Activins A and B have been reported to play important roles in glucose metabolism by regulating the differentiation and activity of the insulin-producing *β* cells and the response of insulin target cells [14, 15]. Administration of follistatin inhibits the differentiation of pancreas duct epithelial cells into *β* cells [16]. The activins, activin B in particular, also have been shown to stimulate insulin gene transcription [15]. On the other hand, activin A increases blood glucose level by reducing target tissue sensitivity to insulin [14]. Moreover, activin A affects inflammation in adipose tissue by stimulating macrophages to switch from a proinflammatory phenotype to an anti-inflammatory phenotype [17]. Thus, obesity-induced inflammation may provide a bridge between T2D and activin activity. In addition, activation of inflammation through the toll-like receptor (TLR) 4 pathway can stimulate release of activin A [12]. TLR4 mRNA and protein expression and TLR4 signaling were increased in recently-diagnosed T2D and TLR4 levels were positively correlated with the glucose level and severity of insulin resistance in this population [18]. 

As a consequence of their multiple roles, the activins could provide an important link between inflammation, glucose metabolism and T2D. Notably, follistatin is correlated with insulin resistance in patients with polycystic ovary syndrome [19]. In order to identify potential roles for the activins and follistatin in onset and established T2D, the following study examined these proteins, as well as several crucial functional parameters, in a small population of subjects with normal glucose tolerance, impaired fasting glucose (IFG), and/or impaired glucose tolerance (IGT), and in patients with T2D. These data indicated a positive relationship between the activins and clinical parameters of T2D, indicating that more detailed investigation of the role of the activins in T2D could be of considerable value.

## 2. Materials and Methods

These studies were approved by the Human Research and Ethics Committee, Monash Medical Centre, Melbourne and adhered to the principles of the Declaration of Helsinki.

### 2.1. Pilot Study

In order to investigate whether acute changes of glucose and insulin levels affect the levels of the activins and follistatin in serum, five healthy men aged 25–50 years were given a standard oral glucose tolerance test (OGTT) with 75 g glucose in 300 mL water. Venous blood samples were collected at 0 min, 60 min and 120 min [20]. 

### 2.2. Main Study

Participants were recruited through advertisement from the community and ambulatory care clinics. Inclusion criteria for participants were as follows: between 50 to 75 years of age, postmenopausal for at least six months, nonsmokers for at least one year, and no changes to prescribed medications in the previous three months. Participants were excluded if they had type 1 diabetes mellitus, previous arterial surgery, known renal impairment (eGFR < 60 mL/min), unstable angina, class 3 or 4 New York Heart Association congestive heart failure, severe peripheral vascular disease (rest pain or active ulceration), presence of inflammatory diseases (e.g., rheumatoid arthritis), or regular usage of nonsteroidal anti-inflammatories or prednisolone. After informed consent was obtained in writing from each participant, all participants underwent a baseline assessment that included a medical history, blood pressure measurement, calculation of body mass index (BMI) and waist: hip ratio (WHR). BMI was calculated as weight in kilograms divided by the square of height in metres. All subjects had fasting serum and plasma (in EDTA) samples and urine samples collected on the morning of study. Subjects were advised to avoid caffeine-containing drinks during a 12 hour overnight fast prior to the study. An OGTT was administered to the participants who had no known history of T2D [20]. Venous blood samples were collected at 0 min, 60 min and 120 min [20]. Participants underwent only fasting blood collection if they had a known history of T2D.

### 2.3. Lifestyle Factors

Lifestyle factors were annotated to include any history of smoking, the level of exercise (score 1 ⩽ 1 hour/week, 2 = 1–3 hours/week, 3⩾3 hours/week), and alcohol intake (score 1 = never, 2 = 1 drink/week, 3 = 1-2 drinks/week, 4 = 3-4 drinks/week, 5 = 5-6 drinks/week, 6 = daily drinking).

### 2.4. Biochemical and Immunoassay Measurements

Activin A was measured by a two-site ELISA (Oxford Bio-innovations, Cherwell, Oxfordshire, UK), using human recombinant activin A as standard [21]. This assay measures both free and follistatin-bound activin A dimers and has no significant cross-reaction with other isoforms of activin, such as activin B. The mean assay sensitivity was 12 pg/mL. The mean intra-assay and interassay coefficients of variation (CVs) were 9.5% and 6.3%, respectively.

Activin B was measured by a two-site ELISA (Oxford Bio-innovations, Cherwell, Oxfordshire, UK), using human recombinant activin B as standard [22]. This assay measures both free and follistatin-bound activin B dimers and has no significant cross-reaction with other isoforms of activin. The mean assay sensitivity was 12 pg/mL. The mean intra-assay and interassay coefficients of variation (CVs) were 6.7% and 4.1%, respectively.

Follistatin was measured by radioimmunoassay (RIA) [23]. This assay uses human recombinant follistatin (supplied by the National Hormone and Peptide Program, NHPP) as standard and tracer. The mean assay sensitivity was 0.97 ng/mL. Mean intra- and interassay CVs for follistatin were 9.4% and 8.4%, respectively. 

All other clinical measurements were performed by the clinical laboratory of the Monash Medical Centre Pathology Department). Plasma glucose was measured with the glucose oxidase method. Insulin was measured using the Access/DXI Ultrasensitive one-step immunoenzymatic assay (Beckman Coulter Diagnostics Australia). Glycated haemoglobin (HbA1c) was measured via a cation exchange column, based on high performance liquid chromatography (TOSO Corporation). Total cholesterol, high density lipoprotein (HDL) cholesterol, and triglycerides were measured using a commercial enzymatic assay (Beckman Coulter Diagnostics Australia). Low density lipoprotein (LDL) cholesterol was calculated using the Friedewald equation [24]. Serum creatinine was measured with an automated colorimetric method (Beckman Coulter SYNCHRON LX20PRO, Sydney, Australia) using a modified kinetic Jaffe reaction. Renal function was expressed as estimated glomerular filtration rate (eGFR) based on the Modification of Diet in Renal Disease study [25]. High sensitivity C-reactive protein (hsCRP) was measured using a Near Infrared Particle Immunoassay (Beckman Coulter Diagnostics Australia). Urinary assays were corrected for individual urinary creatinine levels. 

Homeostasis model assessment of insulin resistance (HOMA-IR) was estimated with the following formula: insulin resistance = fasting plasma insulin (in microunits per ml) × fasting plasma glucose (FPG, in millimoles per litre)/22.5. Patients were assessed as normal if HOMA-IR was <2.5, and insulin-resistant if HOMA-IR was ≥2.5) [26]. Area under the curve for OGTT glucose levels (AUCg) was calculated using trapezoidal integration [27].

### 2.5. Statistical Analyses

Continuous data are reported as mean ± standard deviation and categorical data as percentages. Mean comparisons were made by independent samples *t*-test and analysis of variance (ANOVA), as appropriate. Pearson correlations were used to investigate the associations between the activins, follistatin, and confounding variables, including age, history of smoking, BMI, systolic blood pressure (SBP), diastolic blood pressure (DBP), mean arterial pressure (MAP), pulse pressure (PP), centrally-derived systolic blood pressure (cSBP), use of medications, and diabetes clinical parameters. Multivariate models were subsequently generated by multiple linear regression analysis to identify independent relationships between the activins, follistatin and the clinical parameters. All statistical tests were two-sided and *P* values of less than 0.05 were considered statistically significant. All statistical analyses were performed using SPSS version 19 (SPSS Inc., Chicago, US) or GraphPad Prism 5 (GraphPad, San Diego, US). 

## 3. Results 

### 3.1. Subject Characteristics

Thirty-nine subjects had a normal OGTT, 17 subjects had impaired fasting glucose/impaired glucose tolerance (IFG/IGT), and 36 subjects had T2D according to the American Diabetes Association (ADA) and WHO criteria (Table 1) [28, 29]. Amongst the 36 subjects with T2D, 24 were on oral hypoglycaemic medication, either metformin or sulphonylureas, or both. On average, subjects in the T2D group were older and had higher WHR, HbA1c, AUCg, fasting glucose, fasting insulin, triglycerides, HOMA-IR and lower total cholesterol, HDL and LDL levels than those in the normal group, and were more likely to be using anti-hypertensive medication. There were no differences between the groups in level of exercise or current alcohol consumption, history of smoking, BMI, Crcl (creatinine clearance), and HsCRP (*P* > 0.05, not shown). 

### 3.2. Responses to Acute Changes in Glucose and Insulin Levels in Normal Subjects

**Figure 1:**
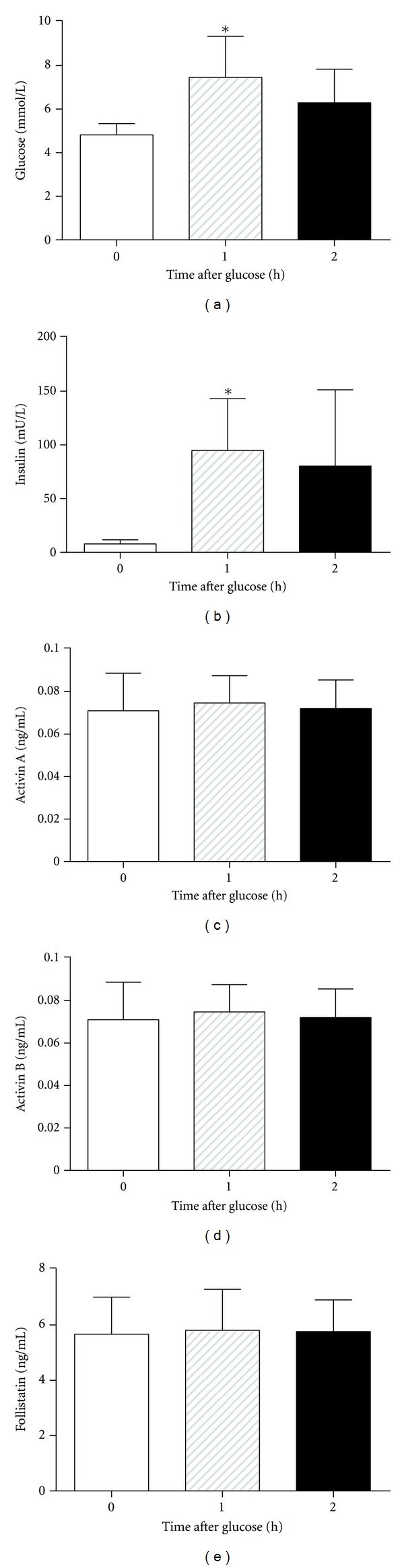
Blood levels of (a) glucose, (b) insulin, (c) activin A, (d) activin B, and (e) follistatin during an OGTT of normal participants (pilot study). All data are mean ± SD, *n* = 5 subjects. *P < 0.05 compared with 0 h value.

Acute changes of glucose and insulin levels following an OGTT did not affect the concentrations of activin A, activin B or follistatin in the circulation of normal participants in the subsequent 2 h period (Figure 1). 

### 3.3. Correlations of the Activins and Follistatin with General Subject Characteristics

In the total study population (*n* = 92), serum levels (mean ± SD) of activin A, activin B, and follistatin were 93.3 ± 27.0 pg/mL, 81.8 ± 30.8 pg/mL, and 6.52 ± 3.15 ng/mL, respectively. There were no significant differences in serum activin A, activin B, follistatin, and HsCRP between the normal, impaired glucose tolerance (IFG/IGT), and T2D groups in the subject population (Table 1), although there was clear evidence of a trend towards an increase in both activins and follistatin in the T2D group compared with the other two groups.

Serum levels of activin A, but not activin B or follistatin, were positively correlated with age in normal subjects and the IFG/IGT group, but not in the T2D group (Tables 2–4). Activin A and follistatin, but not activin B, were also significantly correlated with BMI and past history of smoking in T2D subjects.

Activin A and B and follistatin showed no association with gender, WHR, alcohol consumption, level of exercise, serum cholesterol, HDL, LDL, triglycerides, or anti-glycaemic medication used in any group (*P* > 0.05, data not shown), with the following exceptions: activin B showed a weak negative correlation with HDL in total subjects (*r* = − 0.224, *P* < 0.05), and follistatin showed a negative correlation with HDL in normal subjects only (*r* = −0.364, *P* < 0.05) and a positive correlation with triglycerides in the T2D group only (*r* = 0.410, *P* < 0.05).

Activin A, but not activin B or follistatin, was also significantly elevated in subjects with a history of hypertension (*P* < 0.001). In a parallel study by some of the authors (M. Wu et al., submitted manuscript), activin A, but not activin B or follistatin, was found to be positively correlated with the following blood pressure variables: cSBP, PP, and MAP. There was a positive relationship between activin A levels and the use of ACE inhibitors (*r* = 0.347, *P* < 0.001), beta blockers (*r* = 0.355, *P* < 0.001) and/or diuretics (*r* = 0.344, *P* < 0.001), but not with the use of statins, calcium-channel blockers or aspirin (*P* > 0.05). Activin B was significantly elevated in subjects using diuretics only (*P* < 0.01), and follistatin was not affected by any of these medications.

### 3.4. Correlations of the Activins and Follistatin with Functional Parameters of T2D and Insulin Resistance

**Figure 2:**
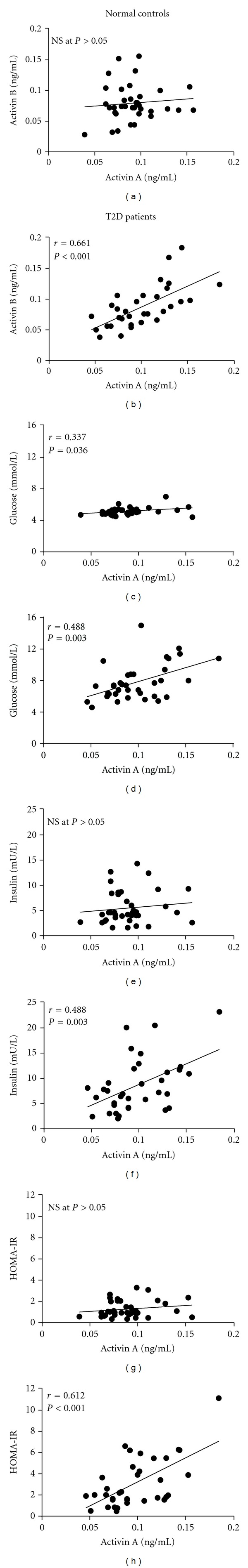
Individual subject data for the correlations of blood levels of activin A with activin B (a, b), glucose (c, d), insulin (e, f), and HOMA-IR (g, h) in normal controls and T2D patients. Note that this figure presents some data from Table 2 in graphical form.

There was no relationship between activin A and B in normal subjects or in the IFG/IGT group, but activin A levels overall were positively correlated with activin B as a result of a very strong relationship within the T2D group (Figure 2 and Table 2). Neither activin A nor activin B showed a correlation with follistatin in any group (Tables 2 and 3).

Activin A and B were correlated with most functional parameters of T2D and insulin resistance (fasting glucose, fasting insulin, HbA1c, and HOMA-IR, although not AUCg) among total participants and in the T2D group, specifically, with the single exception that activin B was not significantly correlated with fasting insulin in the T2D group (Tables 2–4). Activin A and B were also correlated with fasting insulin and HOMA-IR in the IFG/IGT group. After adjusting for covariants/confounding factors (including age, BMI, history of smoking, history of hypertension and medication use, blood pressure parameters), activin A lost its correlation with HbA1c but retained a positive association with fasting glucose, fasting insulin and HOMA-IR, due almost entirely to very strong correlations in the T2D group (Figure 2). After adjustment, activin B lost its correlation with fasting insulin, but retained a positive association with fasting glucose, HbA1c and HOMA-IR. Follistatin was positively correlated with HOMA-IR and AUCg among total participants only, and this was not altered after multivariate analysis.

Follistatin was positively correlated with HsCRP levels in all the groups of subjects, but neither activin A nor activin B showed significant correlation with HsCRP (Tables 2–4). Neither activin A nor activin B was significantly correlated with the kidney function parameters, Crcl and eGFR (data not shown), whereas follistatin was positive correlated with Crcl (Tables 2–4).

## 4. Discussion 

This study examined the levels of activin A, activin B, and follistatin in the blood of normal subjects, and in patients with IFG/IGT or T2D. Despite circulating concentrations of these proteins not being different between the three groups, significant correlations between the levels of these proteins and several functional parameter of T2D and insulin resistance were noted, such as fasting glucose, fasting insulin, HbA1c, and HOMA-IR. The data suggest that a much larger sample population should produce significant differences between the mean activin and follistatin levels in normal and T2D patient groups. A number of other correlations were also apparent. This differs from a previous study in patients with acute myocardial infarction whereby activin A was elevated in the group with higher blood glucose levels, but is consistent with a study showing that blood activin A levels were not altered in T2D subjects compared with control subjects [30, 31]. The difference might be due to the different inflammatory conditions involved, since acute myocardial infarction causes acute inflammation and oxidative stress, whereas type 2 diabetes is a chronic inflammatory disorder [2, 30]. Although the serum levels of CRP, commonly used as a marker of inflammation, were not significantly increased in the T2D groups, it should be noted that an increase in CRP is not a universal feature of T2D [32, 33]. This suggests that inflammation may not be the major factor leading to the T2D observed, at least in this particular set of subjects. In the present study, however, a significant relationship between hsCRP and follistatin, but not the activins, was observed. 

The correlations are consistent with a previous study which showed activin B subunit mRNA expression in adipose tissue was positively correlated with adipose and serum fasting insulin level [34]. These observations of a positive correlation between activin A or B or follistatin and T2D and insulin resistance parameters indicates that the activins might be a clinical indicator for the severity of T2D, which may not only indicate the glucose control condition but also reveal the severity of insulin resistance, and that larger clinical trials of this relationship are definitely warranted.

The results also show that activin A is positively correlated with age in all the participants except the T2D group, which is consistent with our data showing that in a healthy population, and activin A is elevated with increasing age [35]. While activin B has no correlation with age [22]. This is also the first time activin B and follistatin have been examined for a relationship with past smoking. Our study shows that none of anti-diabetic medications affect the level of activins or follistatin, although previous data showed that metformin was able to reduce activin A release from monocytes *in vitro* [31]. Aspirin, likewise, was not significantly correlated with any of the glucose parameters, which is different from data in rats showing that aspirin is able to improve the clinical glucose and insulin resistance parameters in T2D [10]. Finally, this study showed that activins A or B or follistatin were significantly correlated with cardiovascular disease risk factors, such as obesity (BMI), smoking, and lipid profiles (HDL and triglyceride).

The fact that serum levels of activins or follistatin are not clearly predictive of T2D or insulin resistance in individual patients could be due to the complicated roles of activin A and B in the regulation of glucose and insulin metabolism. As previously described, activin A and B both have the ability to improve insulin resistance and help release of insulin to control the blood glucose, but they also promote inflammation in the insulin-responsive tissues and contribute to insulin resistance [14]. This study also suggests that activin A and activin B were not changed with acute changes of glucose and insulin. This differs from a previous study in patients with acute myocardial infarction whereby activin A was elevated in the group with higher blood glucose levels, but is consistent with a study showing that blood activin A levels were not altered in T2D subjects compared with control subjects [30, 31]. This can be explained by the fact that activins are inflammatory factors which would be elevated in an inflammatory response, whereas acute changes of glucose or insulin levels will not cause inflammation in normal subjects. This is also the first clinical study to measure activin B in glucose metabolism, whereas previous studies have examined activin A in glucose metabolism, and follistatin in insulin resistance [19, 30, 31].

The correlations between the activins, follistatin, T2D, and insulin sensitivity indicate a complex set of mechanisms is involved. Activin A and B promote *β*-cell differentiation, insulin gene expression, and insulin secretion from *β* cells [14, 15]. Conversely, in the insulin-responsive tissues, liver, skeletal muscles, and adipose tissue, activin shrinks the size of the organ thereby reducing insulin sensitivity [14]. The effect of activin A on glucose metabolism might also be due to one of the nuclear receptors, such as peroxisome proliferator-activated receptor (PPAR)*γ*, which improves insulin sensitivity by increasing peripheral glucose disposal and decreasing hepatic glucose production [36]. It has been shown that activin A is able to inhibit the expression of PPAR*γ* [37]. Further, activins' contribution to insulin resistance could also be the consequence of activation of one of the adipokines, plasminogen activator inhibitor 1 (PAI-1). In obese subjects, increased expression of PAI-1 in adipose tissue is associated with insulin resistance [38]. After binding to activin receptors, activins activate Smad intracellular signaling molecules, and Smad 3 and 4 are able to enhance the expression of PAI-1 in adipose tissue, one of the insulin target tissues [39]. As a result, activin A and B might increase insulin resistance by stimulating expression of PAI-1. A further mechanistic consideration is that free fatty acid (FFA) levels, which are increased in T2D and central obesity, might play an important role in driving activin and FS changes in T2D. FFA is able to stimulate Toll-like receptor 4 (TLR4), a key signaling molecule in the innate immunity pathway, which is also known to stimulate the release of activin A [1, 12].

In conclusion, this study indicates that measurements of activin A, B, or follistatin cannot discriminate risk for T2D in individual patients, but nonetheless suggests that the activins and follistatin might have important roles in insulin resistance and the onset and development of T2D. Clinically, activin A, B, or follistatin may be useful as indicators of the severity of T2D and insulin resistance. Furthermore, the tissue specific manner of activins's function of modulating insulin sensitivity and insulin secretion may help identify the target tissue for prevention and management of T2D and insulin resistance. Therefore, further studies to investigate the role of the activins, as well as its binding protein follistatin, in this disease may be valuable for the development of future diagnostics or therapeutics. 

## Figures and Tables

**Table 1:** Subject characteristics.

	Normal (*n* = 39)	IFG/IGT (*n* = 17)	T2D (*n* = 36)	*P* value
Age (years)	57.0 ± 5.8	58.0 ± 7.1	62.6 ± 8.4	<0.05
Male gender (%)	38	35	64	—
WHR	0.88 ± 0.08	0.88 ± 0.07	0.95 ± 0.07	<0.001
BMI (kg/m^2^)	27.4 ± 5.5	28.3 ± 3.1	30.0 ± 4.8	NS
History of smoking	13	8	16	—
Fasting glucose (mmol/L)	5.2 ± 0.5	5.7 ± 0.5	7.8 ± 2.3	<0.001
HbA1c (%)	5.6 ± 0.3	5.9 ± 0.3	7.3 ± 0.9	<0.001
Fasting insulin (mU/L)	5.5 ± 3.2	6.2 ± 2.5	8.6 ± 5.4	<0.001
HOMA-IR	1.3 ± 0.77	1.6 ± 0.66	3.1 ± 2.4	<0.001
AUCg^†^	872.0 ± 132.3	1075.5 ± 147.8	1399.5 ± 205.6	<0.001
History of hypertension	7	6	28	—
Total Chol (mmol/L)	5.2 ± 0.8	5.2 ± 1.0	4.4 ± 1.0	<0.001
LDL (mmol/L)	3.3 ± 0.77	3.1 ± 0.9	2.6 ± 0.9	<0.05
HDL (mmol/L)	1.5 ± 0.4	1.4 ± 0.4	1.1 ± 0.2	<0.001
TG (mmol/L)	1.0 ± 0.5	1.6 ± 0.7	1.5 ± 0.9	<0.05
Crcl (mL/min)	97.0 ± 33.4	99.2 ± 30.0	94.8 ± 40.6	NS
HsCRP (ng/mL)	3.0 ± 4.5	2.1 ± 3.8	2.7 ± 4.8	NS
Antihyperglycaemic use	0	0	24	—
Antihypertensive use	5	3	14	—
Activin A (pg/mL)	90.1 ± 24.8	89.6 ± 20.3	98.4 ± 31.6	NS
Activin B (pg/mL)	79.5 ± 29.5	76.1 ± 27.5	87.3 ± 33.8	NS
Follistatin (ng/mL)	6.2 ± 2.4	7.0 ± 2.5	6.7 ± 4.1	NS

WHR: waist to hip ratio; BMI: body mass index; HbA1c: glycated hemoglobin; HOMA-IR: homeostasis model assessment of insulin resistance; AUCg: area under the curve for OGTT glucose levels; SBP: systolic blood pressure; DBP: diastolic blood pressure; Chol: cholesterol; LDL: low density lipoprotein; HDL: high density lipoprotein; TG: triglycerides; Crcl: creatinine clearance; HsCRP: human sensitive C-reactive protein. ^†^Note that only four patients in the T2D patients group were given an OGTT, as most of these patients had a prior diagnosis of T2D. Data are mean ± SD. One way ANOVA was used for analysis. NS: *P* > 0.05.

**Table 2:** Associations between circulating levels of activin A and demographic and biochemical indices within subject groups.

	Total subjects	Normal	IFG/IGT	T2D
Age	0.376***	0.329*	0.550*	0.320
BMI	0.346***	0.227	0.362	0.434**
Smoking	0.205*	0.183	−0.250	0.352*
Fasting glucose	0.388^∗∗∗,†^	0.337*	−0.044	0.488^∗∗,†^
HbA1c	0.386**	0.086	0.084	0.588**
Fasting insulin	0.399^∗∗∗,†^	0.123	0.589*	0.488^∗∗,†^
HOMA-IR	0.485^∗∗∗,†^	0.177	0.547*	0.612^∗∗∗,†^
AUCg	−0.070	−0.009	0.059	−0.195
Crcl	0.045	−0.076	−0.030	0.168
HsCRP	0.110	−0.004	0.380	0.138
Activin B	0.430***	0.099	0.428	0.661***
Follistatin	0.196	−0.028	0.280	0.291

BMI: body mass index; HbA1c: glycated hemoglobin; HOMA-IR: homeostasis model assessment of insulin resistance; AUCg: area under the curve for OGTT glucose levels; Crcl: creatinine clearance; HsCRP: human sensitive C-reactive protein. Data presented as univariate Pearson correlation coefficients (*R*) value: *P < 0.05, **P < 0.01, ***P < 0.001. Correlations with no superscript are not significant (*P* > 0.05). ^†^After adjustment for confounding factors (including age, BMI, history of smoking, history of hypertension and medication use, blood pressure parameters) by multivariate analysis, activin A retained a positive association with fasting glucose, fasting insulin, and HOMA-IR but lost its correlation with HbA1c.

**Table 3:** Associations between circulating levels of activin B and demographic and biochemical indices within subject groups.

	Total subjects	Normal	IFG/IGT	T2D
Age	0.100	−0.206	0.267	0.159
BMI	0.170	0.092	0.321	0.201
Smoking	0.160	0.029	0.356	0.200
Fasting glucose	0.298^∗∗,†^	0.072	0.080	0.406*
HbA1c	0.331^∗∗,†^	−0.193	0.292	0.529**
Fasting insulin	0.254*	0.051	0.510*	0.293
HOMA-IR	0.332^∗∗,†^	0.056	0.514*	0.426^∗,†^
AUCg	−0.100	0.066	−0.155	−0.274
Crcl	0.009	0.098	−0.121	0.004
HsCRP	0.120	0.021	−0.243	−0.119
Follistatin	0.026	0.008	−0.010	0.033

BMI: body mass index; HbA1c: glycated hemoglobin; HOMA-IR: homeostasis model assessment of insulin resistance; AUCg: area under the curve for OGTT glucose levels; Crcl: creatinine clearance; HsCRP: human sensitive C-reactive protein. Data presented as univariate Pearson correlation coefficients (*R*) value: *P < 0.05, **P < 0.01. Correlations with no superscript are not significant (*P* > 0.05). ^†^After adjustment for confounding factors (including age, BMI, history of smoking, history of hypertension and medication use, blood pressure parameters) by multivariate analysis, activin B retained a positive association among total subjects with fasting glucose, HbA1c, and HOMA-IR but lost its correlation with fasting insulin. In the T2D group, only HOMA-IR retained a positive correlation with activin A after adjustment.

**Table 4:** Associations between circulating levels of follistatin and demographic and biochemical indices within subject groups.

	Total subjects	Normal	IFG/IGT	T2D
Age	0.028	−0.041	0.399	−0.054
BMI	0.200	0.004	0.140	0.373*
Smoking	0.304**	0.162	0.226	0.413*
Fasting glucose	0.130	−0.221	0.172	0.175
HbA1c	0.100	−0.061	0.274	0.073
Fasting insulin	0.180	0.082	0.304	0.187
HOMA-IR	0.268*	0.041	0.351	0.323
AUCg	0.271*	0.071	0.411	−0.042
Crcl	0.210	−0.011	0.095	0.371*
HsCRP	0.585***	0.394*	0.642**	0.740***

BMI: body mass index; HbA1c: glycated hemoglobin; HOMA-IR: homeostasis model assessment of insulin resistance; AUCg: area under the curve for OGTT glucose levels; Crcl: creatinine clearance; HsCRP: human sensitive C-reactive protein. Data presented as univariate Pearson correlation coefficients (*R*) value: *P < 0.05, **P < 0.01, ***P < 0.001. Correlations with no superscript are not significant (*P* > 0.05). Follistatin was positively correlated with HOMA-IR and AUCg among total participants only, and this was not altered after multivariate analysis for confounding factors (including age, BMI, history of smoking, history of hypertension and medication use, blood pressure parameters).
